# Phantom for fluorescence uniformity and distortion assessment of near-infrared fluorescence guided surgery systems

**DOI:** 10.1117/1.JBO.30.8.086002

**Published:** 2025-08-18

**Authors:** Emmanuel A. Mannoh, Edwin A. Robledo, Samuel S. Streeter, Ethan P. M. LaRochelle, Alberto J. Ruiz

**Affiliations:** a QUEL Imaging, White River Junction, Vermont, United States; b Geisel School of Medicine, Dartmouth College, Department of Orthopaedics, Hanover, New Hampshire, United States

**Keywords:** fluorescence-guided surgery, fluorescence uniformity, geometric distortion, tissue-simulating phantom, flat-field correction, fluorescence imaging

## Abstract

**Significance:**

The expanding use of fluorescence in surgery necessitates standardized characterization methods to facilitate reproducibility and regulatory review of imaging devices. Current guidelines suggest the use of optical phantoms as tools to quantify optical system performance, yet measurements of uniformity and spatial accuracy or distortion remain challenging and are performed in an ad hoc manner or not collected at all.

**Aim:**

We introduce a photostable solid phantom, the reference uniformity and distortion (RUD) phantom, and accompanying analysis code for characterizing fluorescence uniformity and geometric distortion (GD). In addition, the concept of fluorescence flat-field correction is explored using this phantom.

**Approach:**

The RUD phantom was imaged on a custom fluorescence imaging device, as well as five commercial systems. The analysis code characterized uniformity and distortion in these systems. Flat-field correction was explored on the custom device by imaging solid fluorescent reference phantoms at different locations within the field of view.

**Results:**

Successful characterization of the imaging systems’ uniformity and GD was achieved. Flat-fielding experiments showed that although it qualitatively improves the appearance of images, it could negatively impact quantitative analyses.

**Conclusions:**

The RUD addresses the need for standardized characterization of fluorescence uniformity and GD. Although fluorescence flat-field correction qualitatively enhances image uniformity, caution is advised as it may adversely affect quantitative accuracy.

## 1 Introduction

The use of fluorescence to guide surgery has expanded in recent years since the FDA’s 510(k) clearance of the SPY Imaging System in 2005 for assessing vascular perfusion using indocyanine green (ICG).^1^ In addition to the use of ICG for perfusion assessment, the FDA has also approved fluorescent agents to aid in cancer delineation and resection, such as 5-ALA for brain cancers,^2^ pafolacianine for ovarian^3^ and lung cancer,^4^ and pegulicianine for breast cancer.^5^ With these approvals has also come the proliferation of fluorescence guided surgery (FGS) imaging systems, making it important to have characterization and standardization methods to facilitate FDA review of new devices and allow cross-device comparisons.^6^

A task group formed by the American Association of Physicists in Medicine assembled a list of key metrics needed to characterize the performance of fluorescence imaging systems.^7^ This task group and recent draft guidance by the FDA^8^ highlight the importance of using phantoms for system characterization. Key metrics include: the sensitivity of the system to the fluorophore of interest, the sensitivity of the system to fluorescence at depth, spatial resolution, depth of field, overall fluorescence signal detection uniformity, and geometric distortion (GD). Efforts have been made to develop solid phantoms that enable characterization of these key metrics.^9^^–^^13^ These efforts have largely focused on characterizing sensitivity to fluorophore concentration, sensitivity to fluorescence at depth, and spatial resolution. Although some have addressed signal uniformity, these attempts have generally involved sparse sampling of the fluorescence response across the field of view,^12^^,^^13^ or homogeneous liquid phantoms^14^ that suffer from lack of reproducibility and shelf stability. Furthermore, to our knowledge, there has been no focus on developing phantoms or tools for characterizing distortion in FGS systems.

Fluorescence signal detection uniformity refers to how consistent a fluorescent signal appears across the field of view of the imaging system. It couples both the spatial uniformity of the excitation light source, and the detection uniformity of the camera system (referred to as relative illumination).^15^ Fluorescence uniformity is crucial to characterize when making clinical decisions based on a fluorescence image. For example, in a surgical procedure involving ICG perfusion, a blood vessel may appear well-perfused in the center of the image but poorly perfused when on the periphery of the field of view for systems with significant fluorescence nonuniformity. In such a scenario, the observed difference in fluorescence intensity does not reflect true biological function but rather stems from nonuniformity in the fluorescence signal detection of the imaging system. Although FGS systems have generally been used clinically solely to enhance surgical contrast, recent introductions of semiquantitative assessments, either assigning signal to background ratios in static images or quantifying fluorescence intensity of dynamic flow, also highlight the importance of better fluorescence uniformity characterization.^16^

In an attempt to compensate for this nonuniformity in FGS systems, flat-field correction has been employed.^12^^,^^14^ The term “flat-field correction” comes from digital image processing and involves accounting for spatial nonuniformity in brightness caused by variations in sensor pixel output, imaging optics, and the effects of illumination inhomogeneities.^17^^,^^18^ Although this is an established method in conventional/white light imaging, fluorescence imaging conflates excitation intensity, fluorophore emission, background signals (e.g., autofluorescence), and detector noise into a single measurement, unlike the separable illumination/reflectance in white light imaging.

GD refers to a spatially dependent change in magnification in the image that can alter the perceived shape and size of tissue structures.^7^ Characterizing distortion in FGS systems is equally important as it can cause inaccuracies when estimating the size and shape of anatomical structures or their positions relative to each other.

In this paper, we present a single photostable solid fluorescence phantom, designed to provide assessment of near-infrared (NIR) fluorescence uniformity and geometric distortion. The use of the phantom is demonstrated in custom and commercial fluorescence imaging systems, using open-source analysis code.^19^ Finally, we explore the use of flat-field correction for fluorescence images.

## 2 Methods

### 2.1 Description of the Phantom

**Fig. 1:**
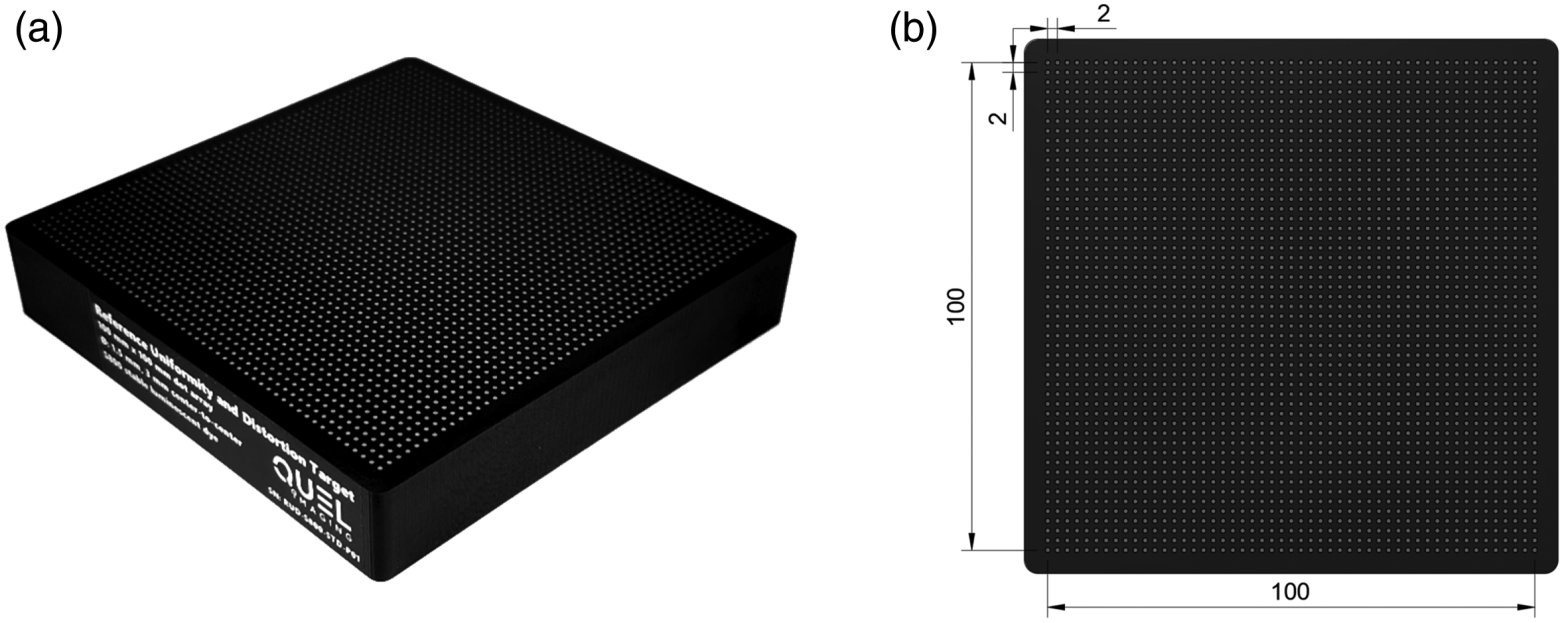
(a) Isometric white-light image of the RUD target and (b) top-down mechanical drawing showing the 100×100  mm imaging area with 1 mm diameter wells with 2 mm center-to-center spacing.

The reference uniformity and distortion (RUD) phantom/target (SKU: RUD_STD_S800-01_TSR-01_r0, QUEL Imaging) are shown in Fig. 1. It is designed for the simultaneous assessment of fluorescence uniformity and geometric distortion. The RUD phantom, manufactured using a proprietary process, consists of a black light-absorbing mold, 3D-printed from photocuring resin that is back filled with a photostable luminescent material. The proprietary luminescent compound (S800-01, QUEL Imaging, White River Junction, Vermont, United States) is broadly excitable in the wavelength range of ∼400 to 790 nm, with a broad emission peaking around 820 nm. The fluorescence excitation and emission spectra of the luminescent material are shown in Fig. S1 in the Supplementary Material. The S800-01 compound is embedded within polyurethane resin. The top surface of the 3D printed mold contains a grid of evenly spaced wells. These wells are filled with S800-01 embedded in the polyurethane resin, enabling the manufacturing of fluorescent dots flush with the top surface of the phantom. The fluorescent wells are each 1 mm in diameter and are spaced at center-to-center intervals of 2 mm over a 100×100  mm imaging area (2601 wells total). The overall dimensions of the target are 110  mm×110  mm×20  mm. The photostability of the luminescent material is demonstrated by comparison with IR-125, the laser-dye form of ICG, under 785 nm excitation at $5\;\mathrm{mW}\;{\mathrm{cm}}^{-2}$ (Fig. S2 in the Supplementary Material).

### 2.2 Obtaining Fluorescence Uniformity Profiles with the RUD Target

Custom Python code was developed to analyze RUD images, generate fluorescence uniformity maps, and quantify imaging uniformity. This code is part of an open-source library, QUEL-QAL, developed to facilitate the analysis of fluorescence reference targets,^19^ available at: https://github.com/QUEL-Imaging/quel-qal (PyPI: quel-qal). If the imaging system field of view is larger than the 100×100  mm imaging area of the phantom, multiple images of the RUD target can be acquired to obtain a fluorescence uniformity profile that spans the field of view (see Fig. S3 in the Supplementary Material for an example where four images were captured to span a ∼110×150  mm field of view). The developed code can stitch the data from these various images together to create a uniformity map that spans the full system field of view. In addition, precise displacements of the target within the field of view can create subsampling of the spatial resolution of the RUD to artificially increase the virtual dot-matrix generated from the combination of multiple acquisition images.

The analysis method is briefly described here. First, the pixel locations and average intensities of the fluorescent wells are extracted from the input image(s) by thresholding and identifying regions of interest (ROIs) based on connected components. The data are then fit to a surface representation using one of two methods: bivariate b-spline interpolation or radial basis function (RBF) interpolation. The b-spline fitting method is the default and produces a smoothed representation of fluorescence uniformity from the input data. The RBF interpolation method is slower and more susceptible to noise but preserves higher-frequency features that the b-spline method would smooth out. The surface representation is normalized so that the maximum fluorescence intensity has a value of 1. Finally, this surface representation can be visualized in multiple ways: a 3D surface plot, a 2D intensity map, line profiles, and a 2D iso-map showing regions of the field of view that are within specified percentages of the maximum fitted fluorescence intensity. Full details on the analysis methods can be found in the QUEL-QAL wiki on GitHub.^20^ Version 0.2.5 of the code was used in analyzing data and producing figures in this study.

#### 2.2.1 Assessment of uniformity quantification

**Fig. 2:**
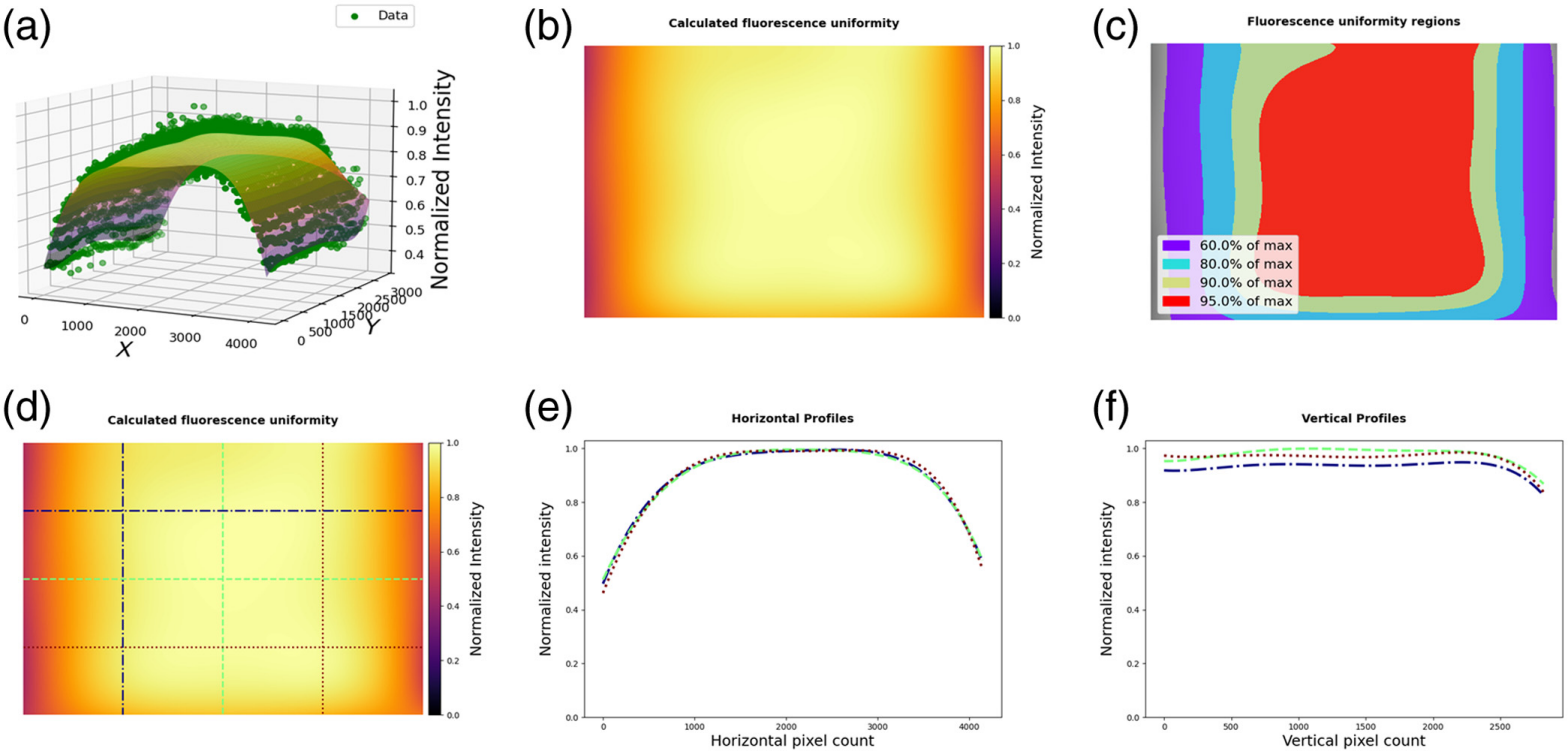
RUD analysis results using b-spline fitting method: (a) 3D plot showing extracted data and fitted surface; (b) fitted fluorescence uniformity map normalized to its maximum; (c) iso-maps, showing regions of the field of view that are at least 60%, 80%, 90%, and 95% of the maximum intensity; (d)–(f) line profiles across the fluorescence uniformity fit.

A custom fluorescence imaging system was used in imaging the RUD target and in subsequent flat-fielding experiments. This system, from here on referred to as the QUEL Imaging Box, has multiple laser excitation wavelengths and emission collection bands. For imaging the RUD target, the excitation wavelength was 760 nm, and the emission was collected with an 805 nm long-pass filter. An example of the visualizations described in Sec. 2.2 produced on this imaging system is shown in Fig. 2.

#### 2.2.2 Photostability and mold material autofluorescence

The photostability of the photoluminescent material and the autofluorescence of the mold material could influence the uniformity measurements. To test the photostability, a 785 nm laser was used to provide irradiation at $5\;{\mathrm{mW}/\mathrm{cm}}^{2}$. A 10 mm diameter sample (20 mm height) was manufactured of the S800-01 material in polyurethane resin. Furthermore, a 10 mm diameter sample (20 mm height) of 300 nM IR-125 in 3D-printed resin (STND-01, QUEL Imaging) was manufactured to serve as a comparison of photostability; the manufacturing method for this sample has been previously published.^21^ A camera with an 800 nm long-pass filter was used to image the wells undergoing the continuous 785 nm irradiation every 90 s. Centered 5 mm ROIs of the wells were used to analyze the fluorescence intensity of each sample at each time point.

Autofluorescence measurements of the mold material were performed on captured images of the RUD target on the QUEL Imaging Box. ROIs of the luminescent wells and mold material were compared with the fluorescence intensity of the ICG concentration sensitivity target described in Sec. 2.7 to assess the material autofluorescence levels. A 760 nm excitation and 805 nm long-pass emission filter was used for imaging. ROIs of half of the well diameters were used for the analysis (5 mm diameter for the 10 mm concentration target wells and 0.5 mm diameters for the 1 mm RUD target wells).

### 2.3 Obtaining Geometric Distortion with the RUD Target

Custom Python code was developed to analyze images of the RUD target to quantify geometric distortion across the imaging system’s field of view. This code is part of the same open-source QUEL-QAL library used for fluorescence uniformity analysis (Sec. 2.2). Full details on the analysis can be found in the QUEL-QAL wiki.^20^ If the imaging system field-of-view exceeds the 100×100  mm imaging area of the phantom, multiple images of the RUD target can be acquired and combined to assess geometric distortion comprehensively across the full system field of view—the only caveat here is that, unlike uniformity assessment, each image must have a minimum number of fluorescent wells identified around the center of the image.

The distortion analysis method is aligned with ISO 17850:2015–GD measurements. Our analysis method is briefly described here. First, the pixel locations of the fluorescent wells are extracted from the image(s) using thresholding and identifying ROIs based on connected components. Next, a regular reference grid is created based on the spacing and orientation of the wells located near the center of the image. Geometric distortion is quantified by comparing the reference grid to the actual measured centroid coordinates of the fluorescent wells (Fig. S4 in the Supplementary Material). Local geometric distortion is calculated using

$$\begin{array}{c} \mathrm{distortion}(\%)=\frac{\text{actual distance}-\text{expected distance}}{\text{expected distance}}\times 100, \end{array}$$ (1)

where actual distance is the distance from the center of the image to each fluorescent well and expected distance is the distance from the center of the image to the corresponding reference grid point from the previous step. The distortion data are then plotted as a function of actual distance from the center of the field of view. In addition to this plot, the distortion data can also be used to produce a 2D map. This map effectively identifies spatial distortion patterns, such as keystone distortion resulting from object or image plane misalignment relative to the optical axis.

#### 2.3.1 Assessment of geometric distortion quantification

**Fig. 3:**
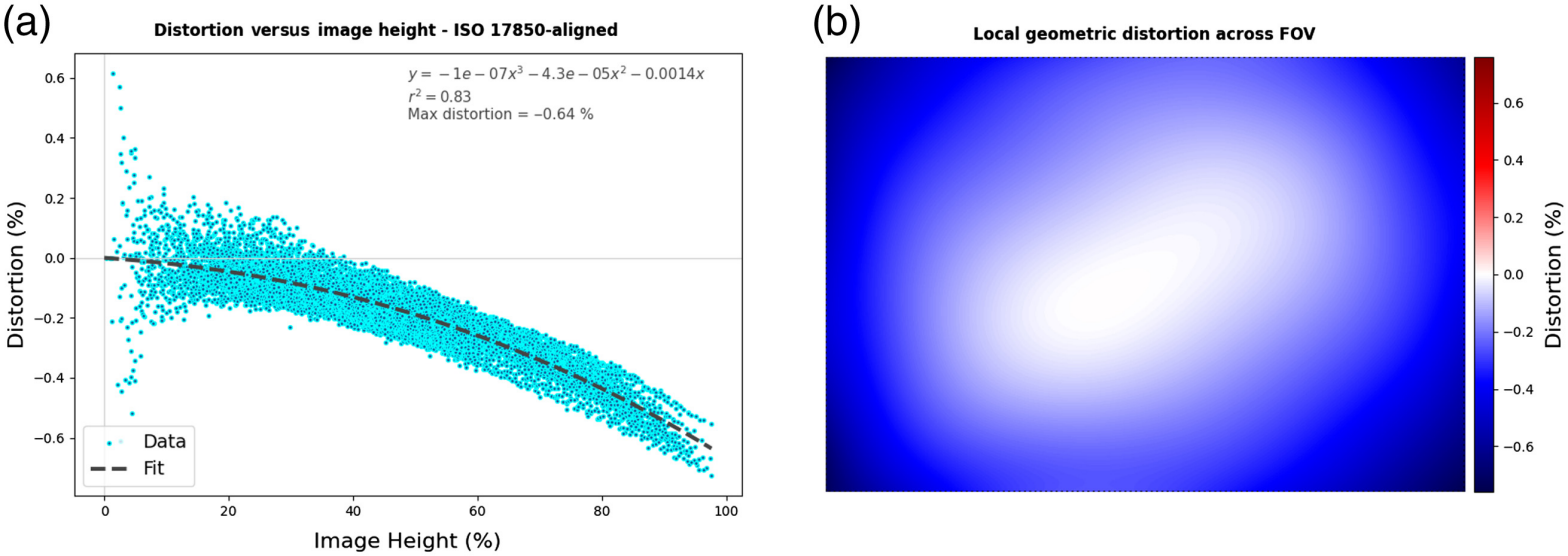
Distortion analysis results: (a) local geometric distortion as a function of image height, showing a small amount of negative or barrel distortion; (b) spatial map of distortion across the field of view, showing radial symmetry.

The QUEL Imaging Box was used to image the RUD target with the same excitation and emission parameters described in Sec. 2.2.1. An example of the distortion visualization described in Sec. 2.3 produced on this system is shown in Fig. 3. To demonstrate keystone distortion, a 3 deg wedge was placed beneath the RUD target, and it was imaged again on the QUEL Imaging Box.

### 2.4 Utilizing the RUD with Commercial Fluorescence Imaging Systems

To explore the variability in fluorescence uniformity and distortion among some commercial fluorescence imaging systems, a RUD target was imaged on five commercial imaging systems at the Dartmouth Hitchcock Medical Center in Lebanon, New Hampshire, United States. The devices were: Pentero 800 S (Zeiss, Oberkochen, Germany), Kinevo 900 S (Zeiss, Oberkochen, Germany), Modus X (Synaptive Medical, Toronto, Ontario, Canada), Pearl Impulse (LI-COR Biosciences, Lincoln, Nebraska, United States), and SPY-PHI (Stryker, Portage, County, Michigan, United States). All systems are state-of-the-art, clinical FGS systems except for the Pearl Impulse, which is a preclinical system. Pearl Impulse imaging data were collected using both the 700 and 800 nm fluorescence channels. The imaging systems are hereafter referred to as system 1, system 2, system 3, system 4A (700 channels), system 4B (800 channels), and system 5, respectively. All systems were capable of imaging the RUD target except for system 5, which has an excitation wavelength of 805 nm^22^ and is beyond the excitation range for the RUD (Fig. S1 in the Supplementary Material). Therefore, it was excluded from further analysis. In all cases, imaging was performed with the room lights off to prevent inadvertent excitation of the RUD target by ambient light. (This measure was not required for system 4 because it is a closed-field imager). For systems that had simultaneous white light and fluorescence imaging, white light illumination was disabled so that the results obtained would truly be representative of just the fluorescence uniformity. This was not possible for systems 1 and 2, and so, imaging was performed with the white light illumination. Each imager was aligned so that its optical axis was orthogonal to the top surface of the RUD target. If a system had zoom capabilities, imaging was performed at the lowest zoom setting to maximize the field of view. For each system, as many images as needed were acquired to span the entire field of view. This required two images each for systems 1 and 2 and one each for the rest. QUEL-QAL code (version 0.2.5) was used to quantify image uniformity and distortion for each system.

### 2.5 Flat-Field Correction: RUD Target

To explore the possibility of using the RUD target for fluorescence flat-field correction, images of the target were first acquired with the QUEL Imaging Box and used to generate a fluorescence uniformity profile. The target was imaged in four locations across the field of view, and at each location, the target was rotated three times by 90 deg (for a total of four imaged orientations). Therefore, in total, 16 images were acquired. The images were analyzed using the analysis method described in Sec. 2.2. For the surface fitting portion, the RBF interpolation method was used because the QUEL Imaging Box has a very structured illumination pattern on the edges of the field of view. After obtaining the fluorescence uniformity profile, the input images were each scaled by the inverse of this profile to produce “flat-field corrected” images. Then, to evaluate the performance of the correction, these flat-field corrected images were passed through the analysis algorithm again, this time using the b-spline method to avoid any high-frequency features or noise. Ideally, the resulting surface should be perfectly flat.

A second set of four images was acquired with the RUD target in only one orientation and spanning the field of view of the QUEL Imaging Box. The purpose of this step was to evaluate flat-field correction on a new set of data that was not used in generating the uniformity profile for the QUEL Imaging Box. These images were scaled by the inverse of the uniformity profile generated above and then passed through the analysis algorithm again using the b-spline method. Ideally, the resulting profile should also be flat.

### 2.6 Flat-Field Correction: Fluorescent Cylinders

For an initial investigation of fluorescence flat-field correction, three 3D-printed cylinders (Ø10  mm) with different concentrations of ICG equivalent fluorophore—100, 3, and 0 nM—were imaged using the QUEL Imaging Box. The tissue-mimicking cylinders had absorption and reduced scattering of 0.021 and $0.27\;{\mathrm{mm}}^{-1}$, respectively, at 800 nm. The manufacturing techniques for these 3D-printed cylinders are described by Ruiz et al.^21^ A custom holder was used to hold the cylinders next to each other and prevent fluorescence bleed-through between them. They were imaged in the following manner. For the first image, the 100 nM cylinder was placed next to the 0 nM cylinder in the center of the field of view. For the second image, these two cylinders were imaged on the left edge of the field of view, where the illumination intensity was known to drop. For the third image, the 3 nM cylinder was placed in the holder next to the 0 nM cylinder in the center of the field of view. For the fourth and final image, the 0 and 3 nM cylinders were imaged on the left edge of the field of view, where the illumination intensity drops.

The average intensity of each cylinder in each image was obtained from a circular ROI that was about two-thirds the radius of each cylinder. All four images were then scaled by the inverse of the fluorescence uniformity profile generated from Sec. 2.5, and the average intensity of each cylinder in each image was measured again. Ideally, after flat-field correction, the intensity of each well should be consistent, regardless of its location in the field of view (i.e., on the edge of the field of view or in the center of the field of view).

### 2.7 Flat-Field Correction: Concentration Sensitivity Target

A reference concentration sensitivity (RCS) target (SKU: RCS-ICG-ST01-QUEL03, QUEL Imaging) was used in this experiment. The target consists of nine wells with varying concentrations of fluorophore (in this case, ICG-equivalent dye) and serves as a dilution series to probe the fluorescence sensitivity of an imaging system.^9^ Here, a total of five images were acquired with this target. First, it was imaged in the center of the field of view, oriented such that the highest concentration well was in the top left. Then, it was imaged on the left edge of the field of view where the illumination intensity drops, in four orientations rotated 90 deg from each other.

The images were analyzed using QUEL-QAL, with the key output metric being the linearity of the intensity-versus-fluorophore-concentration curve. Full details on the analysis methods can be found in the QUEL-QAL wiki.^23^ Briefly, the analysis method quantifies the average intensity of each well using an ROI that is half the diameter of the well, baselines by subtracting the intensity of the control (0 nM) well, normalizes to the maximum, and then fits the resulting data to the equation

$$y=10^{C}x^{m},$$ (2)

where y is the measured ROI fluorescence intensity, C is a constant, x is the fluorophore concentration, and m is a measure of linearity with a value that should ideally be equal to 1.^24^

The images were then scaled by the inverse of the fluorescence uniformity profile from Sec. 2.5 and reanalyzed. If the fluorescence flat-field correction is accurate, the value of m in the corrected images should be closer to 1 than in the uncorrected images.

## 3 Results

### 3.1 Fluorescence Uniformity Profiles

Four images acquired on the QUEL Imaging Box were used as input to the custom Python code for assessing fluorescence uniformity. The analysis results for this system, using b-spline fitting, are shown in Fig. 2. Figure 2(a) shows a 3D plot of the normalized intensity data extracted from the images of the RUD target (green dots) and the fitted surface profile. Figure 2(b) is the fitted surface profile shown as an intensity map and normalized to its maximum. Figure 2(c) displays iso-maps, showing regions of the field of view that have intensity of at least 60%, 80%, 90%, and 95% of the maximum intensity in the fitted uniformity profile. Figures 2(d)–2(f) show horizontal and vertical line profiles taken at evenly spaced intervals (in this case, quarters) across the field of view. Figure S5 in the Supplementary Material shows the same data analyzed using the RBF interpolation method. This RBF analysis result highlights a notable transient dip in intensity at the outer edges of the illumination pattern that was not identified with the b-spline method.

#### 3.1.1 Assessment of photostability and background material autofluorescence

The photostability of the photoluminescent compound was assessed by continuously irradiating the luminescent material for 65 h with a 785 nm laser at $5\;\mathrm{mW}\;{\mathrm{cm}}^{-2}$ (Fig. S2 in the Supplementary Material). After a cumulative dose of $1180\;J\;{\mathrm{cm}}^{-2}$, the fluorescence intensity declined by <2%. Under the same irradiation conditions, the NIR dye IR-125 (laser-grade ICG) exhibited ∼50% loss of fluorescence within just 2 h ($\mathrm{dose}=45\;J\;{\mathrm{cm}}^{-2}$).

To evaluate the RUD mold material autofluorescence, fluorescence images were acquired of the RUD and the ICG RCS target with the QUEL Imaging Box (760 nm excitation, 805 nm long-pass emission); corresponding images and ROI intensity plots provided in Fig. S6 in the Supplementary Material. The mean ROI intensity from the RUD mold material (i.e., autofluorescence) was measured as 5177 counts; by comparison, the RCS control well (0 nM) ROI had a measured signal of 6345 counts. The RUD luminescent wells ROI measured 41,290 counts; by comparison, the 60 and 100 nM RCS target wells measured 31,086 and 51,431counts, respectively. Generally, these results indicate that the mold material has a low autofluorescence signal and provides a suitable low-background substrate for the luminescent wells, which measured signal levels well within the sensitivity range of preclinical and clinical systems.^22^

### 3.2 Local Geometric Distortion

Geometric distortion was quantified using the same images of the RUD target acquired with the QUEL Imaging Box (Sec. 3.1) as input to the custom Python code. The results are shown in Fig. 3. The calculated local geometric distortion becomes more negative with increasing image height (i.e., distance from the center of the field of view), indicating barrel distortion in this system [Fig. 3(a)]. The magnitude of the maximum distortion, however, is less than 1%. In Fig. 3(b), the calculated distortion is displayed as a function of the location of each data point within the field of view. The rotational symmetry of this figure suggests there is minimal, if any, keystone distortion present in this imaging system/setup.

By contrast, when a 3 deg wedge was placed beneath the RUD target prior to being imaged on the same system, the spatial distortion map showed an asymmetry such that the left side of the image had positive distortion, whereas the right side of the image had negative distortion [Fig. S7(b) in the Supplementary Material). In addition, the distortion-versus-image-height plot [Fig. S7(a) in the Supplementary Material] shows two groups of data points, one with slightly positive distortion and the other with more negative distortion.

### 3.3 Fluorescence Uniformity and Distortion of Commercial Devices

**Fig. 4:**
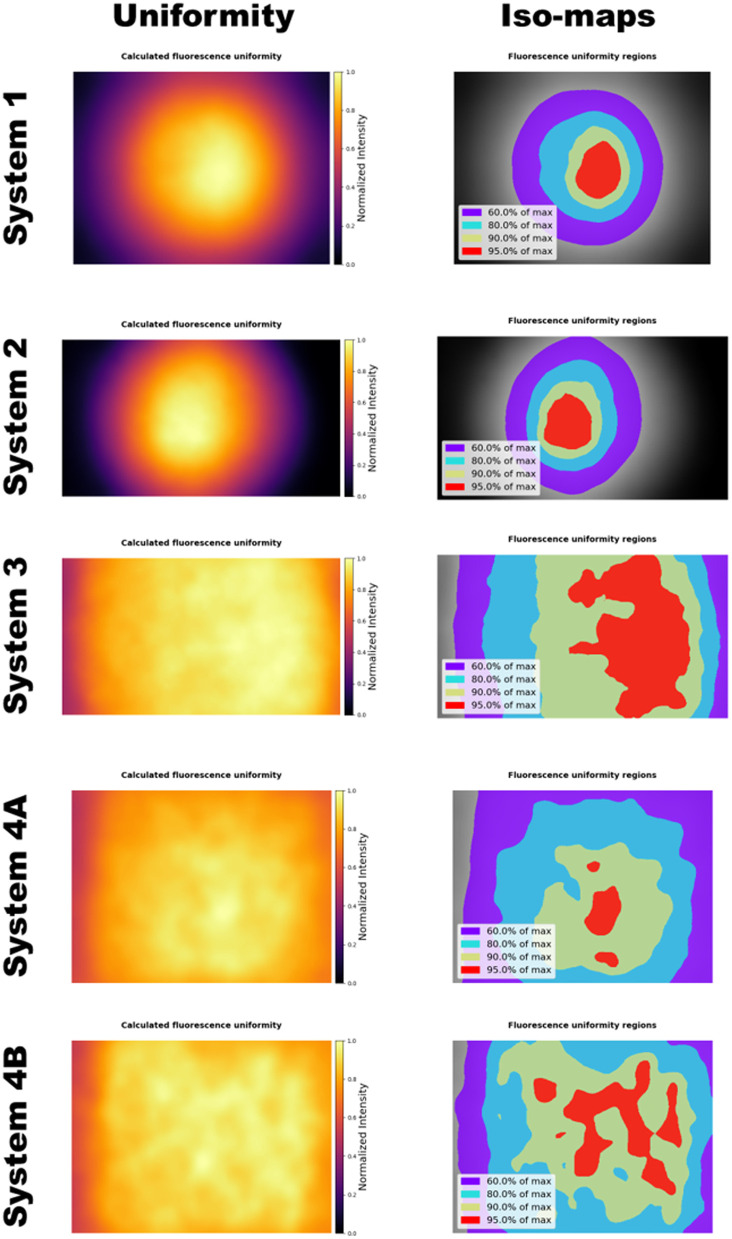
Fluorescence uniformity across the field of view of four commercial fluorescence imaging systems. Systems 3, 4A, and 4B are the most uniform, with almost the entire field of view being at least 60% of the maximum fluorescence intensity. Systems 1 and 2 have much smaller regions where fluorescence intensity is at least 60% of maximum.

**Fig. 5:**
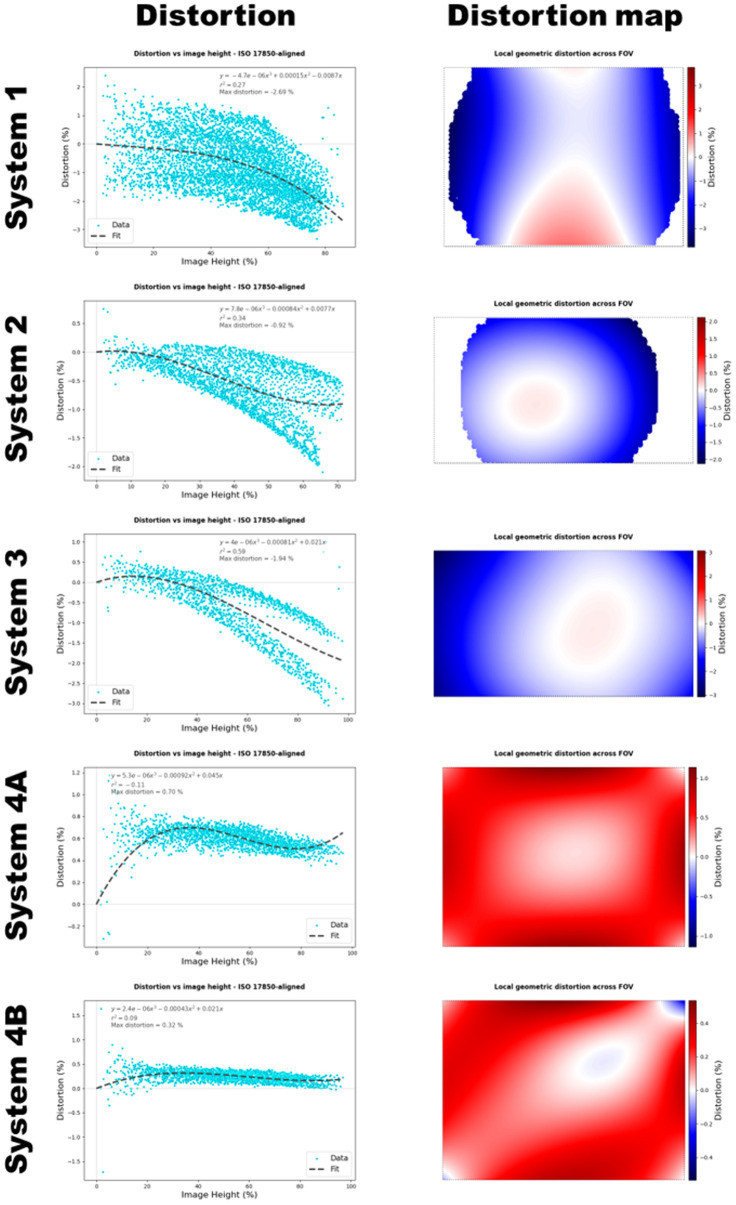
Local geometric distortion across the field of view of four commercial fluorescence imaging systems. System 1 has the most distortion, with a maximum of close to −3%. Systems 4A and 4B have the least distortion of less than 1%.

The results of fluorescence uniformity and distortion assessments for the commercial imaging systems are shown in Figs. 4 and 5, respectively. Systems 3, 4A, and 4B showed the highest fluorescence uniformity, with almost the entire field of view being at least 60% of the maximum fitted fluorescence intensity. By contrast, systems 1 and 2 showed lower uniformity, with less than half of the field of view reaching the 60% threshold. Figure 4 shows the fitted fluorescence uniformity profiles and iso-maps for the systems, generated using the RBF interpolation method.

In terms of distortion, systems 1, 2, and 3 showed some degree of overall negative distortion, whereas systems 4A and 4B had minimal positive distortion. System 1 displayed the greatest distortion, approaching −3%, and had an asymmetric hourglass-like distortion pattern, where localized regions near the top and bottom center of the field of view showed slightly positive distortion. Systems 4A and 4B had the least distortion, both below 1%. The distortion graphs are shown in Fig. 5. System 5 (SPY-PHI, 805 nm excitation) produced weak signal levels from the RUD target due to the low absorption of the S800-01 compound at this excitation wavelength (Fig. S1 in the Supplementary Material), such that no uniformity or distortion metrics could be extracted from the acquired images. Due to the low signal level of these images, they were excluded from further analysis.

### 3.4 Flat-Field Correction: RUD Target

A total of 16 images were used to generate a fluorescence uniformity profile for the QUEL Imaging Box using the RBF interpolation method—the target was imaged at four locations spanning the field of view, in four orientations. The inverse of the resulting profile was multiplied by the input images to produce flat-field-corrected images. An example of one image pre and post this correction is shown in Fig. S8 in the Supplementary Material. These corrected images were then fed back into the custom Python code for assessing uniformity, and the resulting surface was flat within 95% of the maximum value. This is shown in Fig. S9 in the Supplementary Material. A second set of four images was acquired, and these images were corrected with the uniformity profile generated from the 16-image dataset. The resulting images were input to the uniformity assessment code, and the results are shown in Fig. S10 in the Supplementary Material. The resulting uniformity profiles from these images are slightly less flat; however, the entire field of view is again within 95% of the maximum value.

### 3.5 Flat-Field Correction: Fluorescent Cylinders

**Fig. 6:**
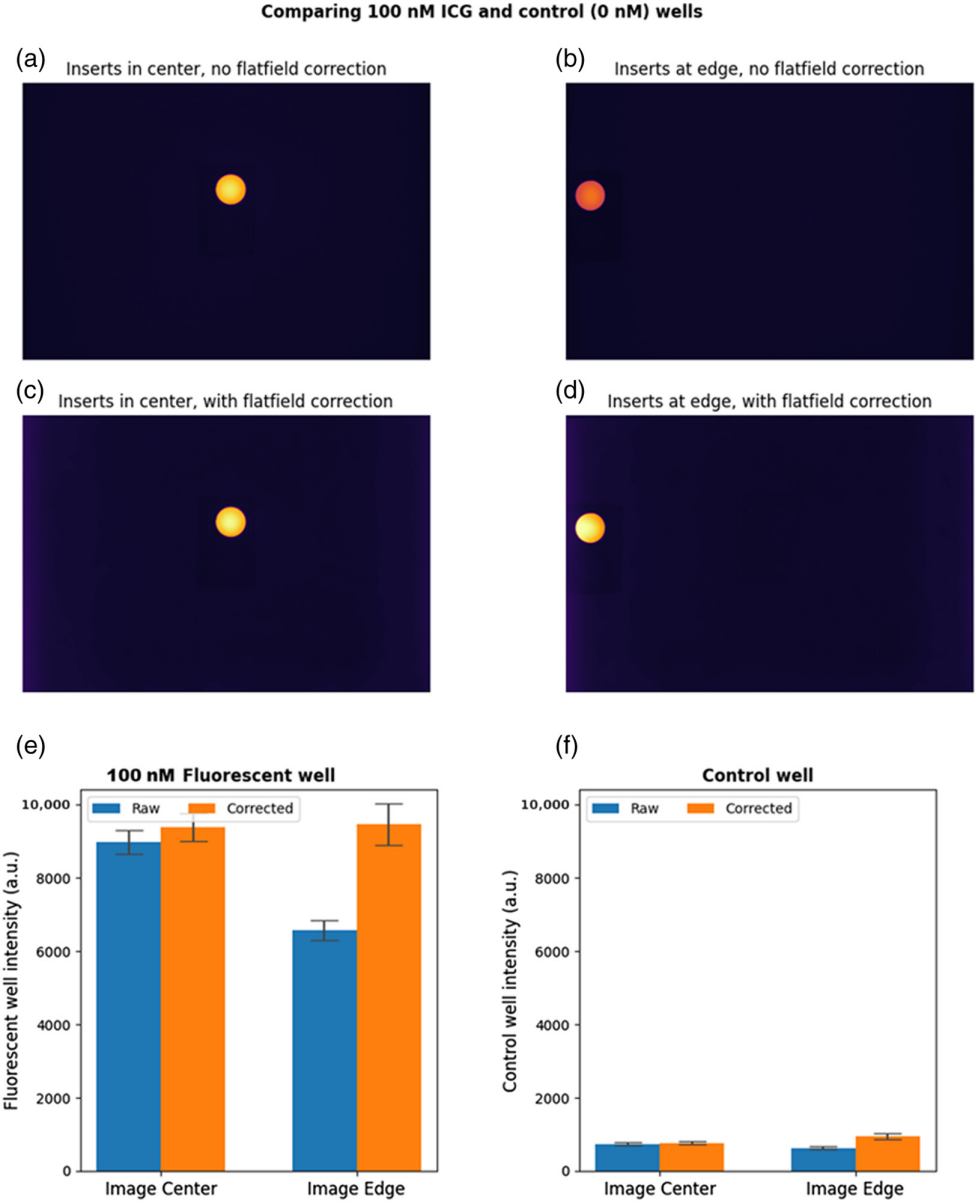
Comparison of 100 and 0 nM fluorescent cylinders with and without flat-field correction: shown are uncorrected images of the fluorescent cylinders in the center (a) and on the edge (b) of the field of view; followed by flat-field corrected images of the cylinders in the center (c) and on the edge (d) of the field of view; and finally, average intensities of an ROI centered on the 100 nM (e) and 0 nM (f) cylinder. When the image is flat-field corrected, the intensity of the 100 nM cylinder on the edge of the image matches its intensity in the center. However, the intensity of the control (0 nM) cylinder on the edge of the field of view overshoots the intensity in the center. Error bars in panels (e) and (f) depict ± 1 standard deviation.

**Fig. 7:**
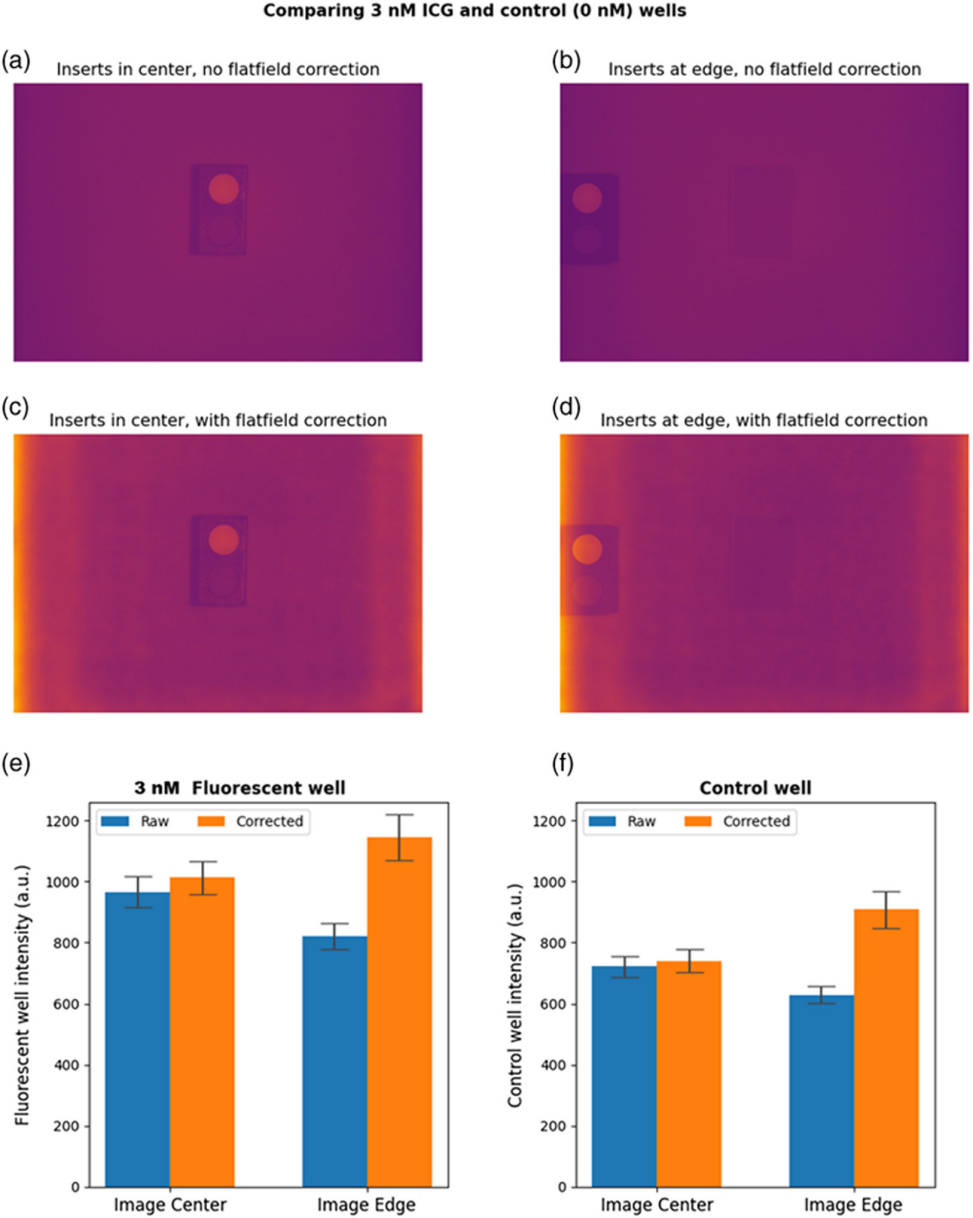
Comparison of 3 and 0 nM fluorescent cylinders with and without flat-field correction: shown are uncorrected images of the fluorescent cylinders in the center (a) and on the edge (b) of the field of view; followed by flat-field corrected images of the cylinders in the center (c) and on the edge (d) of the field of view; and finally, average intensities of an ROI centered on the 3 nM (e) and 0 nM (f) cylinder. When the image is corrected, the intensities of both the 3 nM and control (0 nM) cylinders on the edge of the field of view overshoot the corresponding intensities in the center. Error bars in (e) and (f) depict ± one standard deviation.

Figures 6(a) and 6(b) show the original images of the 100 and 0 nM fluorescent cylinders in the center and on the edge of the field of view. The corresponding images corrected with the uniformity profile from Sec. 3.4 are shown in Figs. 6(c) and 6(d). In Figs. 6(e) and 6(f), the average intensities of the fluorescent cylinders are plotted in bar graphs. The measured ROI averages and standard deviations (fluorescence counts, arbitrary units) for the uncorrected 100 and 0 nM cylinders in the center of the field of view were 8976±326 and 744±37, respectively. The uncorrected values on the edge of the field of view were 6570±277 and 636±28. After flat-field correction, the ROI averages for the 100 and 0 nM cylinders were 9407±354 and 764±38 in the center of the field of view, and 9468±558 and 952±74 on the edge of the field of view. After correction, the intensity of the 100 nM well on the edge of the field of view matches its intensity in the center of the field of view. However, after correction, the intensity of the control (0 nM) on the edge of the field of view is greater than its intensity in the center. Figure 7 shows a similar set of figures for the 3 and 0 nM pairing. Due to the low fluorescence intensity, the influence of the flat-field correction on the nonfluorescent background is more noticeable—the left and right edges of the corrected images have higher intensity than the center. In Figs. 7(e) and 7(f), the average intensities of both the 3 and 0 nM cylinders on the edge of the field of view overshoot their intensities in the center when the images are corrected.

### 3.6 Flat-Field Correction: Concentration Sensitivity Target

**Fig. 8:**
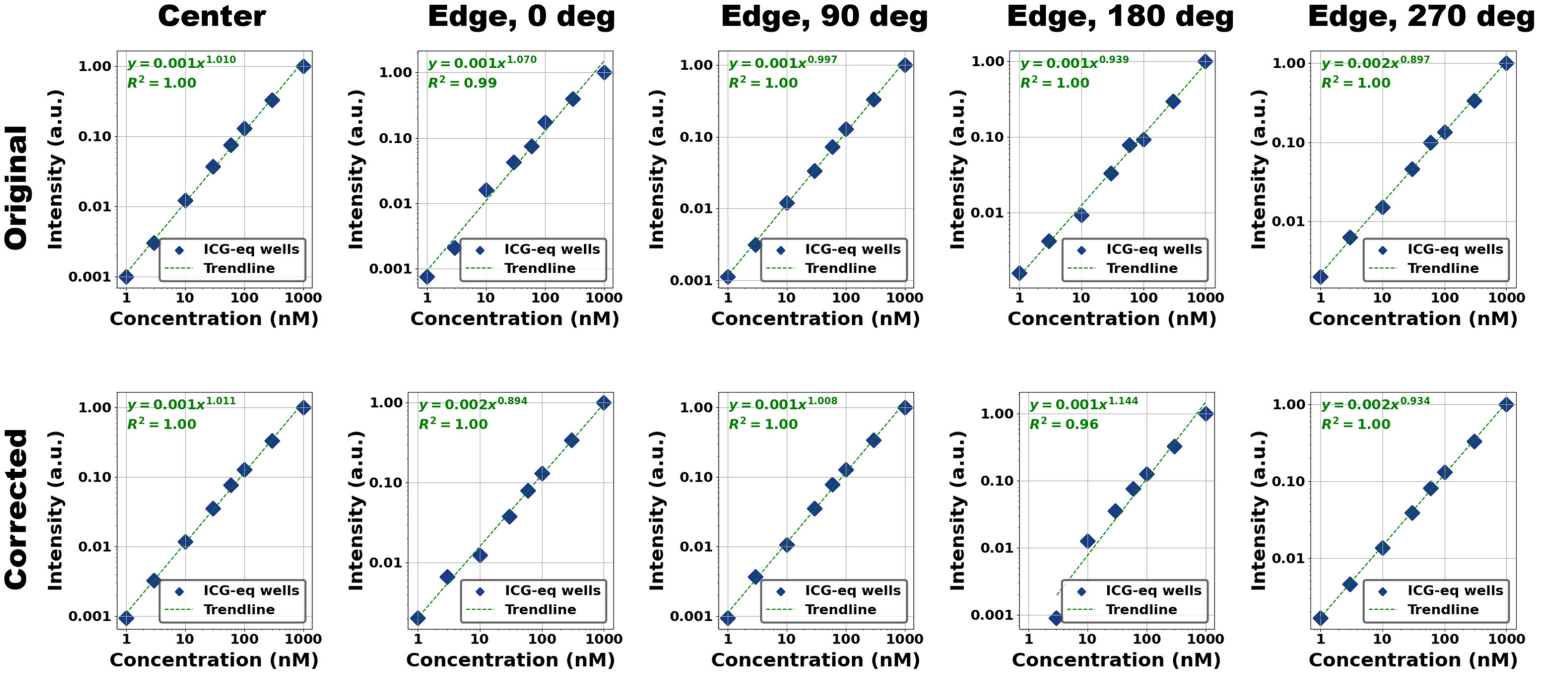
Results from analyzing images of the RCS target pre- (top row) and post- (bottom row) flat-field correction. Each graph is a log-log plot of normalized baselined fluorescence intensity versus fluorophore concentration. Flat-field correction does not generally improve the linearity fit, and in the case of the 180 deg rotation, substantially worsens it.

The acquired fluorescence images of the RCS target are shown in Fig. S11 in the Supplementary Material. The RCS target was images once at the optical center and four times on the left edge of the field of view, each rotated by 90 deg. This placement moves the nine concentration wells from the image center out to ∼50  mm off-axis—well into the region where illumination has already fallen by ∼40% [Fig. 2(c)]—thereby sampling the strongest center–edge nonuniformity. Visually, flat-field correction improves the apparent consistency of fluorescence brightness across the images. Quantitative analysis of the images, however, shows that flat-field correction generally does not improve the measure of linearity, m, and in some cases, worsens it substantially (m should ideally be equal to 1). Particularly, the corrected Edge 0 deg and Edge 180 deg images provide worse linearity metrics. Figure 8 shows these results in log–log plots of normalized baselined fluorescence intensity versus fluorophore concentration. In addition, Table 1 contains the values of m for all five images, before and after flat-field correction. These findings highlight important caveats—namely, potential amplification of background signals and signal-dependent nonlinearity when corrected levels diverge from calibration conditions—and are further addressed in Sec. 4.

**Table 1:** Measure of linearity for RCS target images with and without flat-field correction.

	m				
	Center	Edge, 0 deg	Edge, 90 deg	Edge, 180 deg	Edge, 270 deg
**Original**	1.010	1.070	0.997	0.939	0.897
**Corrected**	1.011	0.894	1.008	1.144^a^	0.934

a For this image, the corrected control well had higher intensity than the corrected 1 nM well, resulting in negative intensity for the 1 nM well after baselining. Hence, it was excluded from the fit to calculate m.

## 4 Discussion

Understanding the spatial uniformity of fluorescence detection across the field of view of a fluorescence imaging system is crucial, especially when clinical decisions depend on fluorescence intensity. Without this characterization, there is a risk of misinterpreting fluorescence images—such as deciding to perform unnecessary additional surgery based on perceived inadequate tissue perfusion during ICG angiography, or failing to identify fluorescently labeled cancerous tissue due to its position in a low-sensitivity region of the imaging field. It is also important to understand the spatial distortion of the imaging system, in particular, for cases where the operator may not have visual access to the imaging subject, such as in endoscopic imaging.

Although the uniformity of fluorescence capture is largely dependent on the illumination source, it is also affected by the spatial light collection efficiency of the optical assembly that detects the signal (i.e., relative illumination). Therefore, to truly assess fluorescence uniformity, simply mapping out the illumination profile, for example, with an optical power meter, is not sufficient. A homogeneously fluorescent phantom, such as a bath of Intralipid into which a fluorophore is diluted, can help capture the fluorescence uniformity profile of an imaging system.^14^^,^^25^ However, it is not shelf-stable and can suffer from a lack of repeatability. Moreover, the risk of contaminating the imaging area with the fluorophore is high, and it cannot measure distortion. To address these limitations, we designed the RUD target, a solid phantom that contains a grid of equally fluorescent photostable wells that enables the characterization of both fluorescence uniformity and local geometric distortion simultaneously.

In this work, the RUD phantom was first evaluated on a custom fluorescence imaging system (QUEL Imaging Box) to demonstrate its ability to characterize fluorescence uniformity and geometric distortion. The analysis results generated with the QUEL-QAL library showed that this system has a rectangular-shaped fluorescence capture profile, with almost the entirety of the field of view being at least 60% of the maximum fluorescence intensity. Using the same image dataset, the geometric distortion across the imaging field was also assessed, revealing mild barrel distortion of less than 1%. Although our distortion analysis is consistent with ISO 17850:2015 standards, strict compliance with this standard requires specific conditions, including precise target alignment and dot size relative to the imaging field. In addition, ISO 17850:2015 was originally developed for standard reflectance-based imaging (primarily white-light systems), and the use of image stitching for fluorescence systems with larger fields of view further deviates from this standard. It is worth noting that precise lateral displacement of the RUD between acquisitions allows subsampling of the spatial resolution (1 mm Ø wells with 2 mm spacing), effectively increasing the apparent density of the fluorescent dot-matrix when multiple images are combined.

The photostability results of the S800-01 material, following 65 h of continuous 785 nm irradiation at $5\;\mathrm{mW}\;{\mathrm{cm}}^{-2}$ ($1180\;J\;{\mathrm{cm}}^{-2}$ total dose), showed an ∼2% increase in signal, which is likely due to fluctuations in laser output rather than changes in fluorescence intensity. By contrast, the IR-125 laser dye exhibited ∼50% signal loss after only 2 h under identical conditions. This level of stability supports the use of the RUD phantom for extended or repeated calibrations without introducing photobleaching-related artifacts. The autofluorescence assessment of the mold material showed signal levels below those of the ICG concentration target control well, indicating that the mold material provides a low-background substrate for the fluorescent wells. Moreover, the signal levels measured for the luminescent wells fall within the typical sensitivity range of preclinical and clinical fluorescence imaging systems indicated for use with ICG and 800 channel fluorophores, supporting their suitability as a reference standard.²¹

Five commercial fluorescence imaging systems were characterized for fluorescence imaging uniformity and geometric distortion using the RUD target, demonstrating the phantom’s versatility across diverse device configurations. The results highlighted the differences in uniformity between these systems, with systems 1 and 2, both surgical microscopes, exhibiting much smaller regions of fluorescence uniformity. However, these results are context-dependent: surgical microscopes typically utilize zoomed-in fields of view, focusing primarily on the image center; by contrast, the images used here were acquired with the field of view fully zoomed out. Improved uniformity for zoomed-in fields of view for surgical microscopes has been previously reported.^26^ System 3 is an exoscope, where fluorescence uniformity is likely more critical.^27^ System 4 is a small-animal imaging box, which would also require higher uniformity across the entire field-of-view. The images acquired by system 5 could not be analyzed for uniformity and distortion metrics due to the low signal generated by the 805 nm excitation wavelength, which exceeds the RUD target’s excitation range of 400 to 790 nm; future work will explore alternative luminescent compound formulations that extend the excitation range beyond 800 nm, enabling RUD target compatibility with such systems at the SPY-PHI. Distortion was generally small for these commercial systems, with system 1 showing the greatest distortion of ∼3%.

Using knowledge of an imaging system’s fluorescence uniformity profile to “flat-field” images is of interest in many applications to improve the qualitative appearance of the images and allow for quantitative analyses.^14^^,^^28^^,^^29^ In this paper, we demonstrated that the RUD target can be used for this application, to capture the fluorescence uniformity profile of the QUEL Imaging Box and then flat-field correct a second set of images of the same target. As shown in Figs. S8–S10 in the Supplementary Material, the corrected initial and second set of images had uniform fluorescence across the entire field of view (well within 95% of the maximum). Nevertheless, subsequent experiments caution the use of fluorescence flat-field correction for quantitative analyses.

In one experiment, a fluorescent 3D-printed cylinder containing 100 nM of ICG-equivalent fluorophore was imaged alongside a control (0 nM) cylinder in the center and then on the edge of the field of view of the QUEL Imaging Box. This 100 nM fluorescent cylinder had a similar fluorescence signal intensity to the RUD target wells when imaged, and therefore, when flat-field correction was performed, the corrected intensity on the edge of the image matched the corrected intensity in the center. By contrast, the corrected intensity of the control (which had no fluorophore) was higher on the edge of the field of view than in the center, highlighting inappropriate amplification of nonfluorescent signals. A similar effect was observed with a lower concentration (3 nM) cylinder, again suggesting incorrect intensity scaling resulting from the flat-field correction.

Further testing with the concentration sensitivity target, to span fluorescence intensities across multiple ICG-equivalent concentrations, showed that flat-field correction improved the linearity of imaged intensity versus fluorophore concentration in only one of five imaging scenarios. In two scenarios, the flat-field correction substantially worsened linearity. As observed in the previous experiment, when the fluorescent intensity being corrected is similar to that of the RUD target, flat-field correction produces good results.

To understand why flat-field correction may negatively impact quantitative analyses, several factors must be considered. At low fluorophore concentrations, fluorescence intensity typically increases proportionally with concentration;^30^ however, practical imaging systems inherently have a detection threshold and a nonzero noise floor,^7^ below which proportionality no longer holds. Ideally, regions with no fluorophore should yield zero fluorescence intensity; however, autofluorescence and system background signals contribute to a baseline intensity that is always present, making it impossible to achieve zero intensity without artificial manipulation such as thresholding or baselining. Consequently, flat-field scaling factors derived from fluorescent signals can inadvertently amplify nonfluorescent background and autofluorescence signals disproportionately,^14^ as observed in our experiments with both fluorescent cylinders and the concentration sensitivity target. These observations are similarly noted in prior work emphasizing the limitations of flat-fielding for quantitative interpretation in intraoperative imaging.^14^^,^^25^ In addition, at higher fluorophore concentrations, effects such as self-quenching can reduce fluorescence efficiency,^31^ further disrupting the linear relationship between concentration and detected intensity. Thus, fluorescence flat-field correction is only reliable under conditions where fluorophore concentration and excitation intensity linearly correlate with the detected fluorescence signal, and background signals such as autofluorescence remain negligible. Alternative correction strategies that decouple background and signal, such as two-component models that first subtract a spatially resolved background map before applying an illumination-gain correction, or hybrid approaches combining detector flat-fielding with concentration-matched reference scaling, may alleviate the observed flat-fielding artifacts.^28^^,^^29^ Although the evaluation of such methods was beyond the scope of this study, future work can explore and benchmark these algorithms against the simple multiplicative correction presented here. However, it is worth noting that, due to the nature of fluorescence imaging—where background, autofluorescence, and fluorescence signals cannot be easily decoupled—these alternative strategies may not be realistically implementable in clinical imaging environments, particularly given tissue heterogeneity and nonplanar geometries.

Considering these limitations, extreme caution should be taken when using flat-field correction in quantitative analyses of *in vivo* fluorescence images particularly because fluorophore concentration, autofluorescence, and background signal variations across the imaging field are typically unknown. For example, in ICG angiography, where fluorescence is used as a measure of tissue perfusion,^32^ a piece of tissue on the edge of the field of view might appear dim because it has very little fluorophore (hence, perfusion) or because of the nonuniformity of the imaging system (needing flat-field correction). Hence, we recommend that fluorescence flat-field correction should only be performed for qualitative improvement of *in vivo* images. For quantitative assessment, it is better to identify the region of the imaging system’s field of view that is within an acceptable level of fluorescence uniformity and to only perform assessments within this region, which is consistent with prior work emphasizing the clinical impact of spatial nonuniformity and recommending center-region analysis for reliability.^26^ The RUD target and associated analysis code introduced in this paper can effectively facilitate this identification.

## 5 Conclusion

We developed a solid, shelf-stable fluorescent phantom (RUD) and accompanying open-source image analysis software (QUEL-QAL) for characterizing fluorescence uniformity and geometric distortion in fluorescence imaging systems. The utility of the RUD phantom and analysis code was successfully demonstrated on a custom-built imaging device and four commercial fluorescence imaging systems. The feasibility of fluorescence flat-field correction using RUD-generated uniformity profiles was also explored. Although flat-field correction qualitatively improved fluorescence image uniformity, it can simultaneously negatively impact quantitative accuracy. Consequently, we advise caution when applying flat-field correction in quantitative imaging applications and, instead of relying on this approach, recommend using uniformity assessment to identify regions of the field of view that meet acceptable uniformity criteria for quantitative measurements.

## Supplementary Material

10.1117/1.JBO.30.8.086002.s01

## Data Availability

The data used in this study are available from the corresponding author upon reasonable request. The code used for the fluorescence uniformity and geometric distortion analysis is part of an open-source library, QUEL-QAL, developed to facilitate the analysis of fluorescence reference targets,^19^ available at: https://github.com/QUEL-Imaging/quel-qal (PyPI: quel-qal).
