# Supplementary material for: Phantom for fluorescence uniformity and distortion assessment of near-infrared fluorescence guided surgery systems
Source: J Biomed Opt. 2025 Aug 18;30(8):086002. doi: 10.1117/1.JBO.30.8.086002 (PMC12360714; doi:10.1117/1.JBO.30.8.086002)
Supplement: Supplementary file 1 [file JBO_030_086002_SD001.pdf]

A Phantom for Fluorescence Uniformity and Distortion Assessment of Near-Infrared Fluorescence Guided Surgery Systems

Emmanuel A. Mannoh[1], Edwin A. Robledo,[1] Samuel S. Streeter[2], Ethan P. M. LaRochelle[1], Alberto J. Ruiz[1]*

[1] – QUEL Imaging, White River Junction, VT 05001 USA

[2] – Department of Orthopaedics, Geisel School of Medicine, Dartmouth College, Hanover, NH 03755 USA

* Alberto Ruiz, PhD: alberto@QUELimaging.com

Supplementary Figures

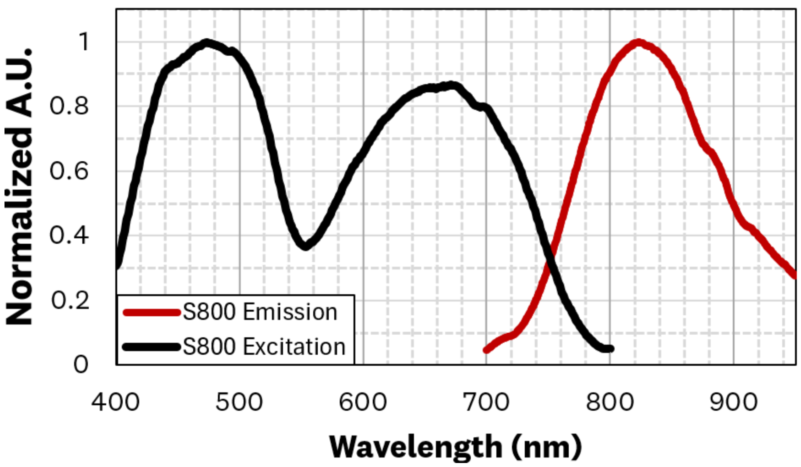

Figure S1. Excitation and emission spectra of the S800-01 luminescent compound

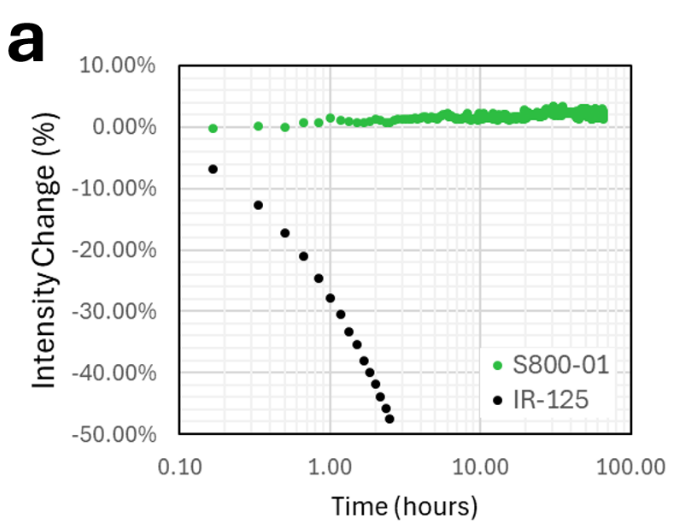

Figure S2. Excitation and emission spectra of the S800-01 luminescent compound. (a) Plot of intensity change vs. time for the 785 nm irradiation (5 mW/cm2) and (b) Plot of the intensity change vs. calculated dose.

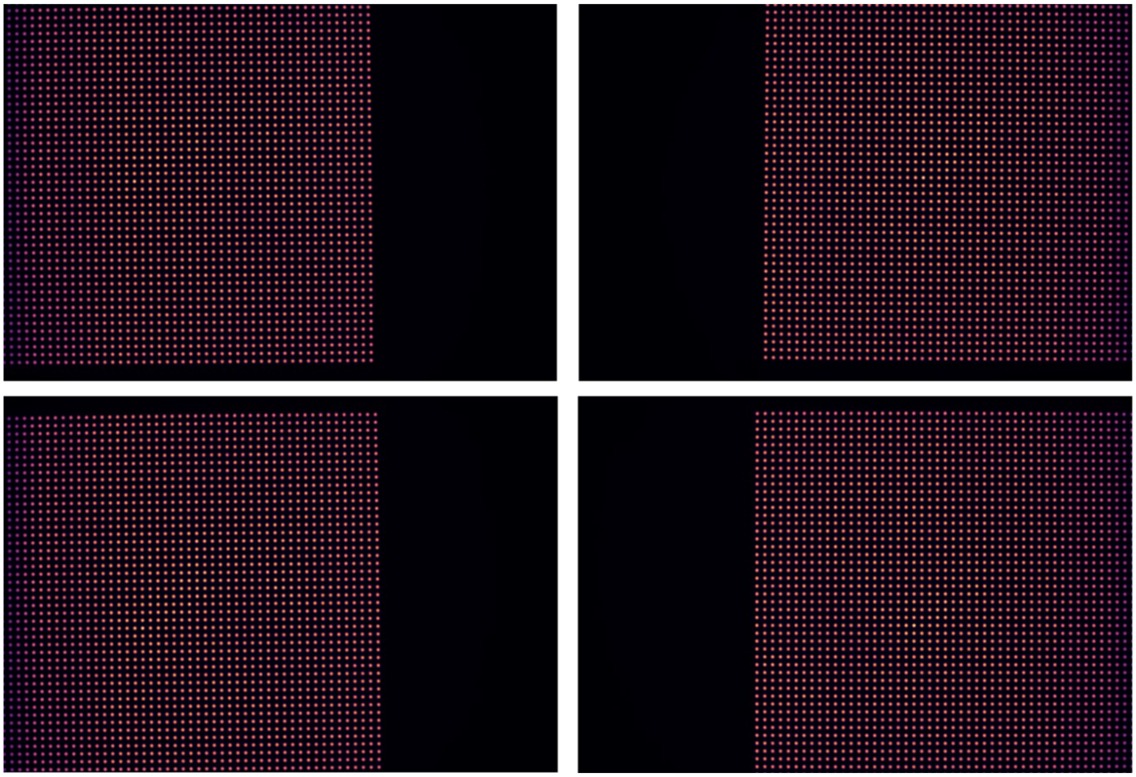

Figure S3. Four fluorescence images of the RUD target, taken so that data from the fluorescent wells span the entire field of view of the imaging system being evaluated.

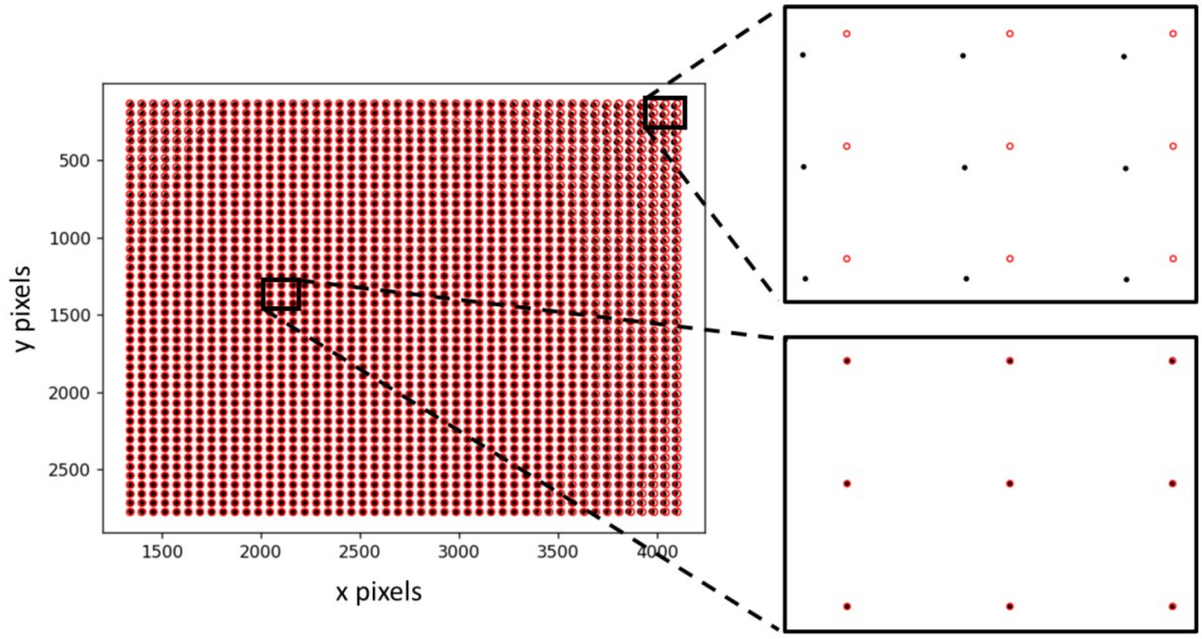

Figure S4. Visualization comparing the imaged well centroids (black circles) to the expected positions (red circles) derived from a regular reference grid. Towards the center of the image, the two overlap, but towards the periphery of the image, they diverge from each other.

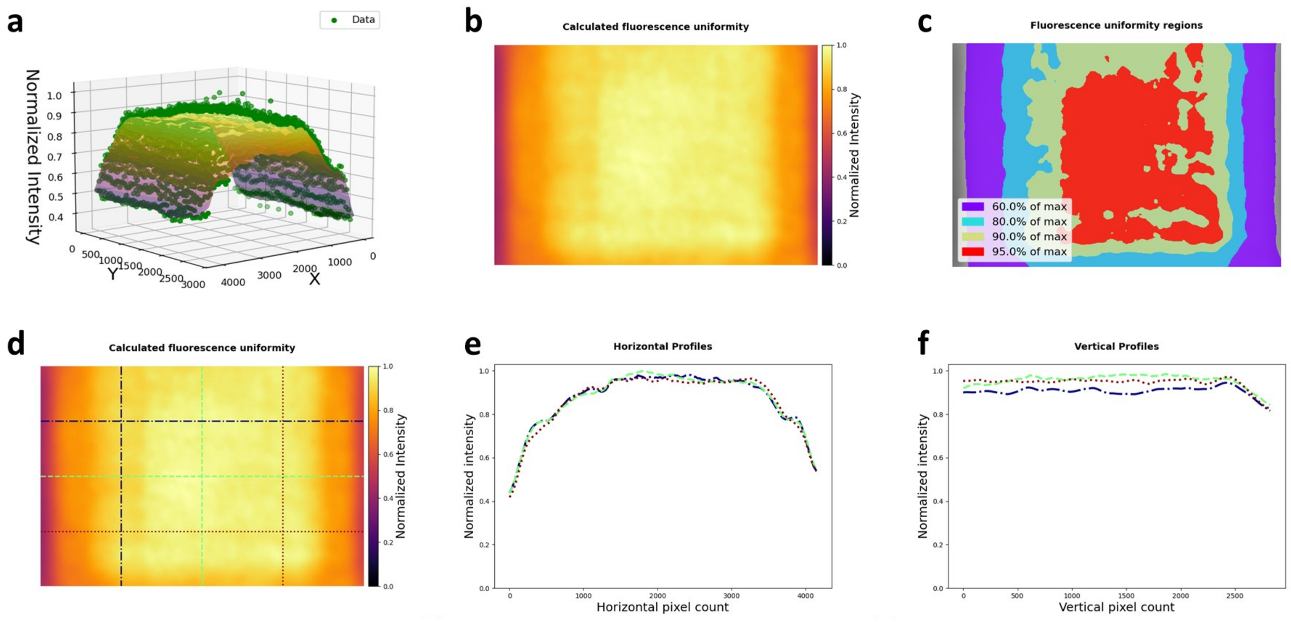

Figure S5. RUD analysis results using RBF interpolation method: (a) 3D plot showing extracted data and fitted surface; (b) fitted fluorescence uniformity map normalized to its maximum; (c) iso-maps, showing regions of the field of view that are at least 60%, 80%, 90%, and 95% of the maximum intensity; (d – f) line profiles across the fluorescence uniformity fit. Note the transient dips in intensity on the outer edges of the uniformity profile that are not present in the results of the b-spline fit.

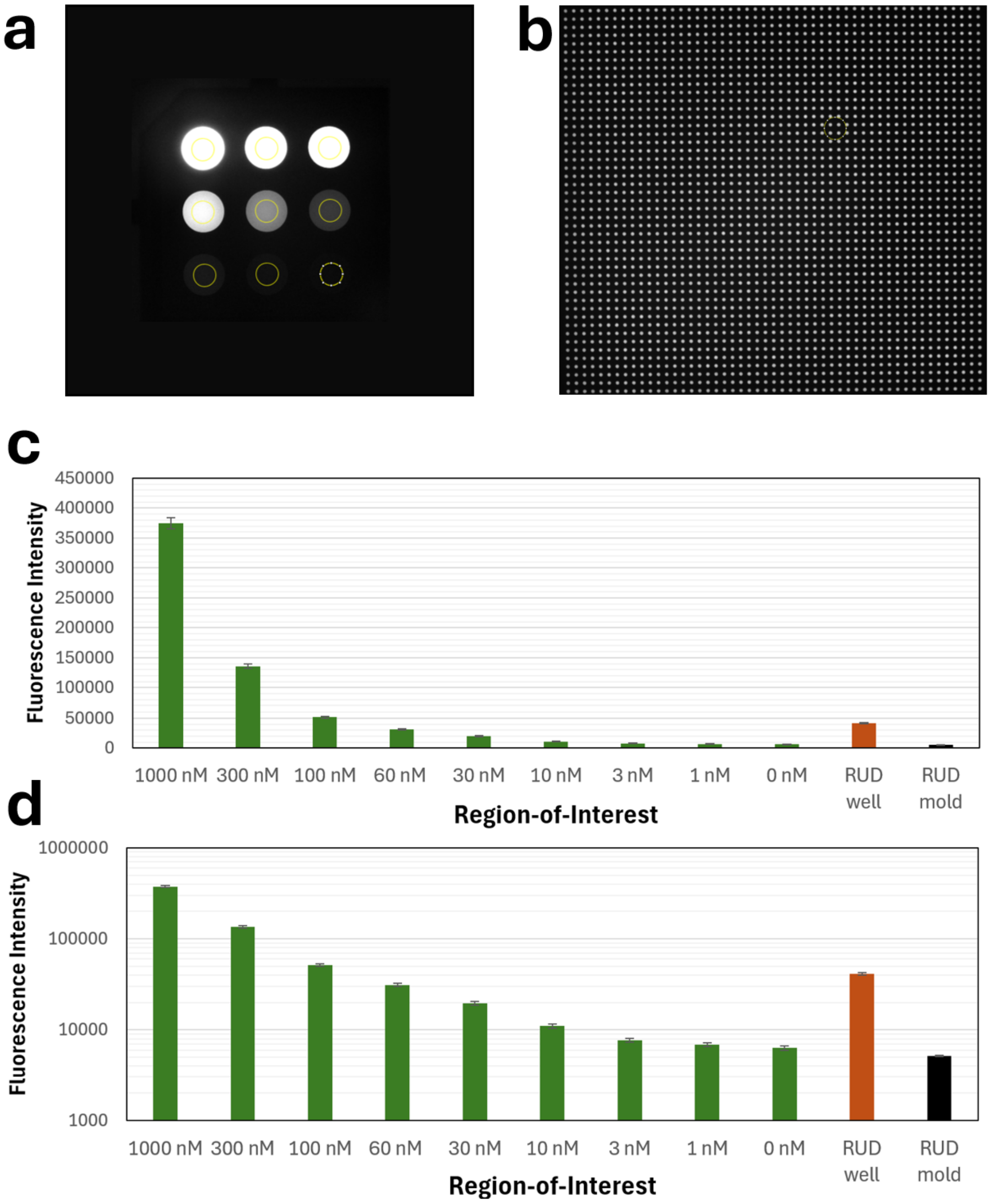

Figure S6. Figure S6. Fluorescence images and region-of-interest (ROI) intensity plots for the ICG concentration target wells (green), RUD fluorescent well (orange), and RUD mold material (black): (a) Fluorescence image of the ICG concentration target, (b) Fluorescence image of the RUD target acquired under identical conditions, (c) Linear-scale ROI fluorescence-intensity plot, and (d) Logarithmic-scale ROI fluorescence-intensity plot. Error bars represent the standard deviation of the corresponding ROIs. A 5 mm-diameter ROI was used for each ICG concentration target well. RUD-well intensity statistics were extracted from four 0.5 mm-diameter ROIs centered on the 1 mm fluorescent wells located within the same imaging area as the 100 nM ICG well (area shown). Fluorescence signal from the RUD mold was measured using four 0.5 mm-diameter ROIs positioned midway between these wells.

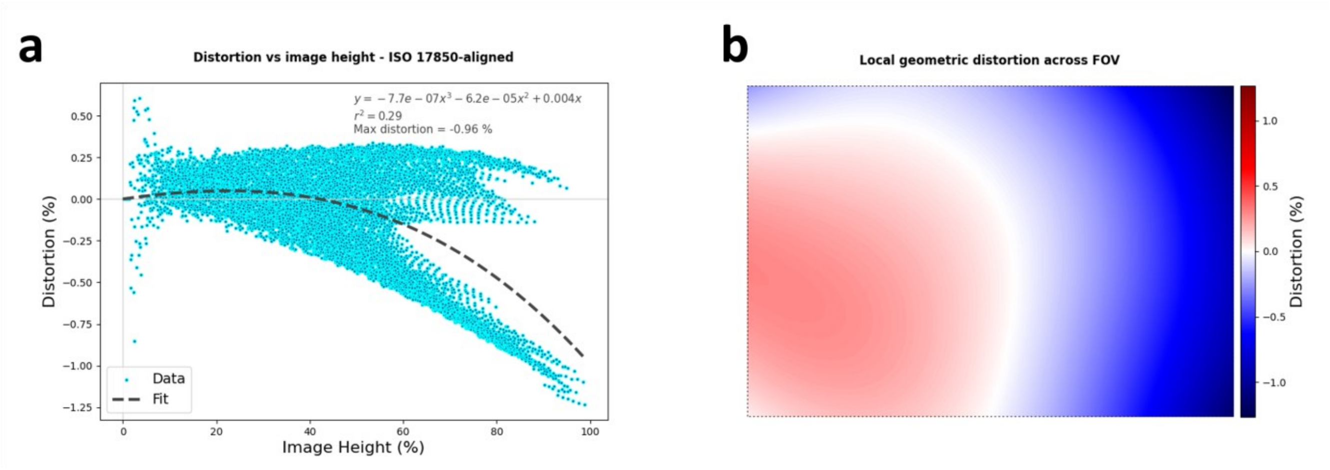

Figure S7. Distortion analysis results after placing 3° wedge underneath RUD target: (a) local geometric distortion as a function of image height, showing two groups of data points, one with slightly positive, and the other with more negative distortion; (b) spatial map of distortion across the field of view, showing clear keystone distortion where the left side of the image has positive distortion and the right side of the image has negative distortion. FOV = field of view.

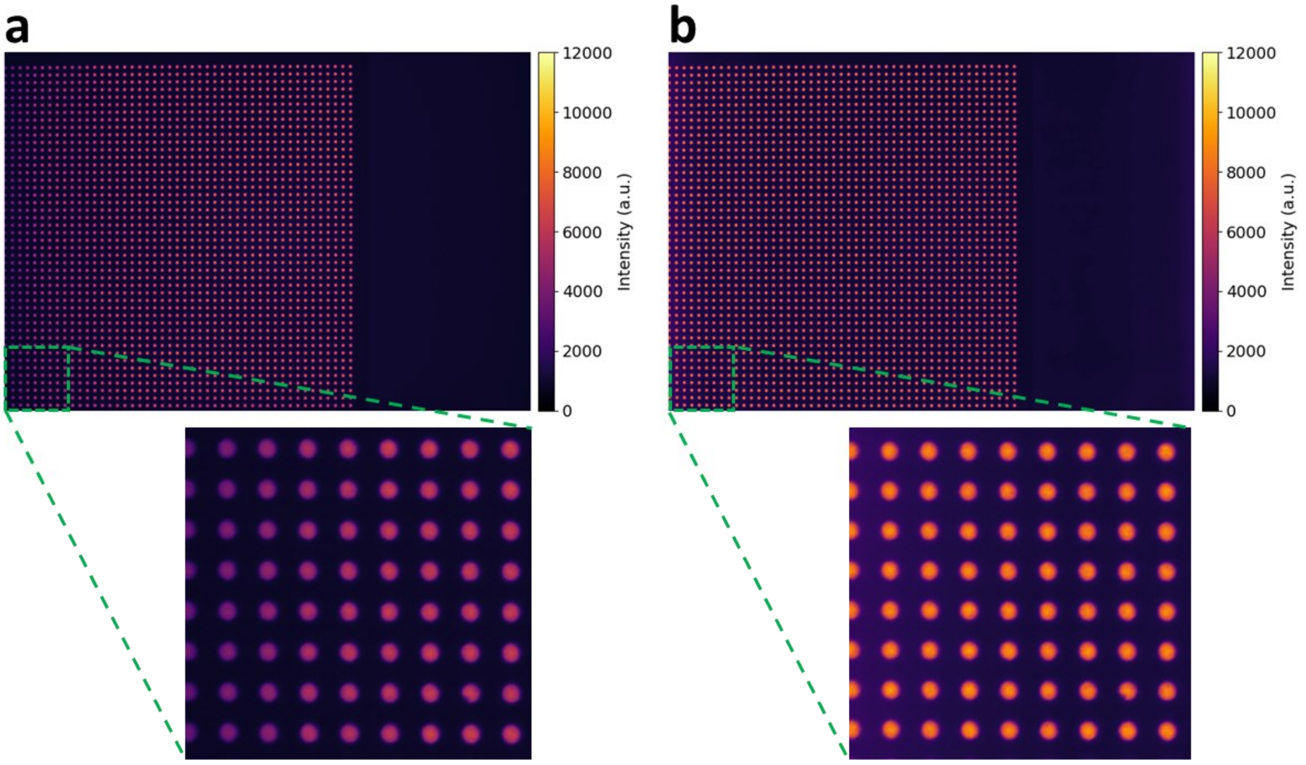

Figure S8. A fluorescence image of the RUD target pre- (a), and post- (b) flatfield correction. The fluorescent wells are more uniform in intensity after the correction.

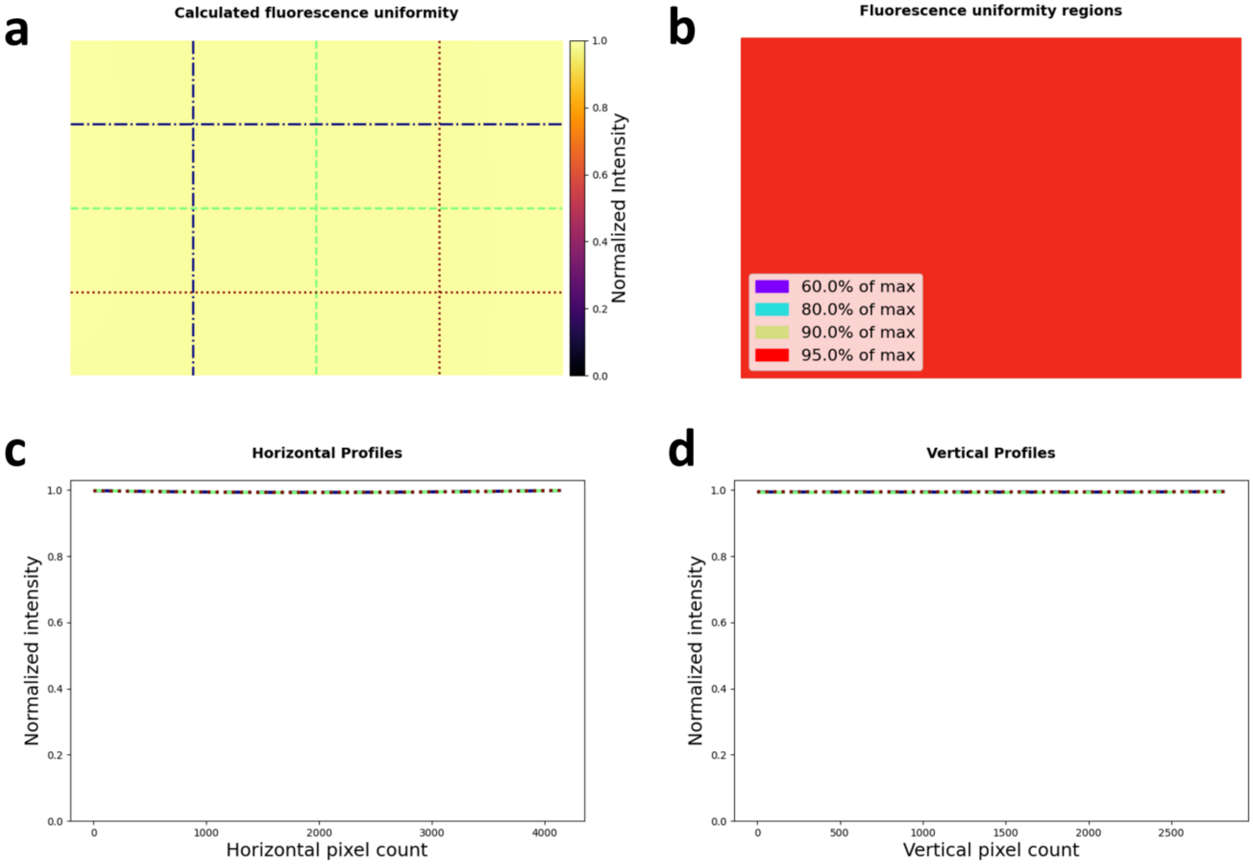

Figure S9. Results from uniformity analysis of flat-field corrected RUD images: line profiles are visually very flat (a, c, d); iso-maps, showing that the entire field of view is at least 95% of the max intensity (b).

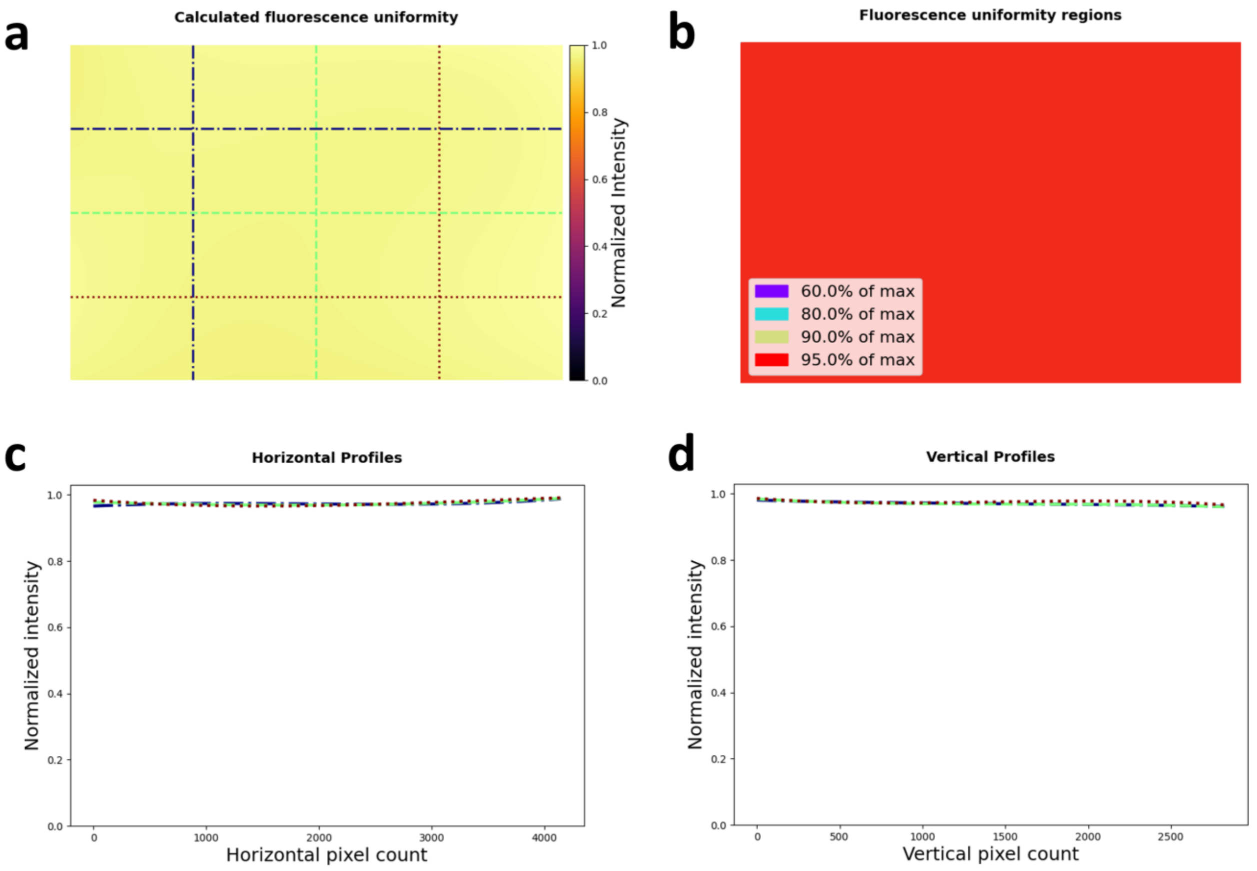

Figure S10. Results from uniformity analysis of new set of flat-field corrected RUD images that were not used in generating the applied uniformity profile: line profiles are a little wavier (a, c, d); however, iso-maps show that the entire field of view is still at least 95% of the max intensity (b).

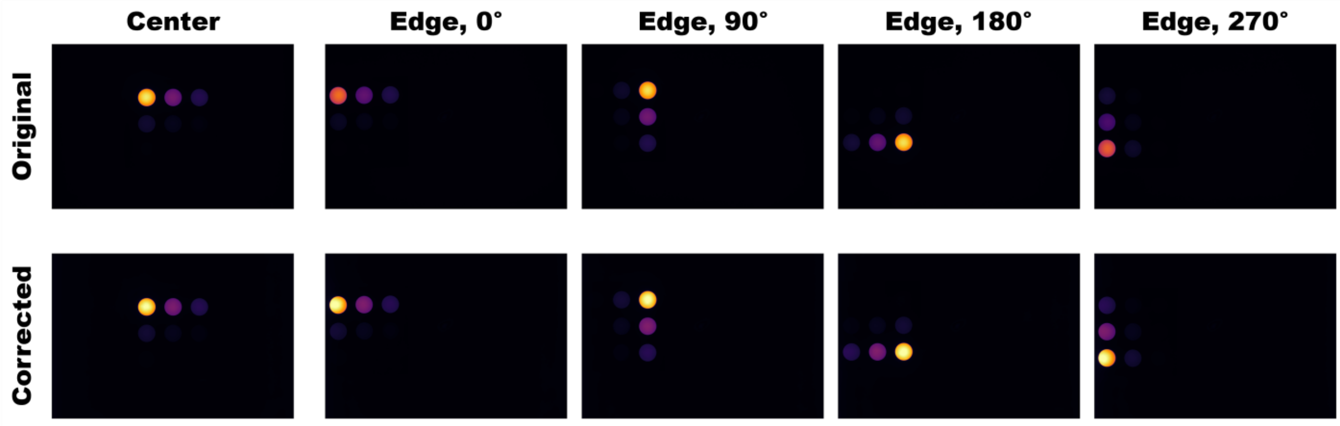

Figure S11. Fluorescence images of the RCS target pre- (top row) and post- (bottom row) flatfield correction. When corrected, the images appear more consistent regardless of orientation of the RCS target or location within the field of view.
